# Metamaterial Absorber for Electromagnetic Waves in Periodic Water Droplets

**DOI:** 10.1038/srep14018

**Published:** 2015-09-10

**Authors:** Young Joon Yoo, Sanghyun Ju, Sang Yoon Park, Young Ju Kim, Jihye Bong, Taekyung Lim, Ki Won Kim, Joo Yull Rhee, YoungPak Lee

**Affiliations:** 1 Department of Physics and RINS, Hanyang University, Seoul, South Korea; 2 Department of Physics, Kyonggi University, Suwon, South Korea; 3 Advanced Institutes of Convergence Technology, Seoul National University, Suwon, South Korea; 4 Department of Display Information, Sunmoon University, Asan, South Korea; 5 Department of Physics, Sungkyunkwan University, Suwon, South Korea

## Abstract

Perfect metamaterial absorber (PMA) can intercept electromagnetic wave harmful for body in Wi-Fi, cell phones and home appliances that we are daily using and provide stealth function that military fighter, tank and warship can avoid radar detection. We reported new concept of water droplet-based PMA absorbing perfectly electromagnetic wave with water, an eco-friendly material which is very plentiful on the earth. If arranging water droplets with particular height and diameter on material surface through the wettability of material surface, meta-properties absorbing electromagnetic wave perfectly in GHz wide-band were shown. It was possible to control absorption ratio and absorption wavelength band of electromagnetic wave according to the shape of water droplet–height and diameter– and apply to various flexible and/or transparent substrates such as plastic, glass and paper. In addition, this research examined how electromagnetic wave can be well absorbed in water droplets with low electrical conductivity unlike metal-based metamaterials inquiring highly electrical conductivity. Those results are judged to lead broad applications to variously civilian and military products in the future by providing perfect absorber of broadband in all products including transparent and bendable materials.

Metamaterials defined as materials which are not existing in nature are artificial materials providing invisible characteristics in the nature, such as negative refraction, perfect absorption, and perfect transmission, through the periodical arrangement of meta-atoms made by the size smaller than incident electromagnetic wavelength by about 1/3–1/5^1^,^2^,^3^,^4^. Because of the particular characteristics, metamaterials can be applied to various fields such as perfect lenses, optical cloaking and microwave antenna^5^,^6^,^7^,^8^. Among them, perfect metamaterial absorber (PMA) which absorb electromagnetic wave with metamaterials can be applied as plasmonic sensor, solar cells, photo-detectors, thermal emitter and thermal imager^9^,^10^,^11^,^12^,^13^. Especially, PMA can block electromagnetic wave which is generated in Wi-Fi, cell phones and home appliances used daily and harmful for body. Furthermore, it can provide stealth function avoiding radar detection to military unmanned aerial vehicle, fighter, tank and warship. While those strengths are announced, people pay more attention to it.

After metamaterial absorber was firstly introduced as plane form patterned by copper on dielectric substrate^11^, various research results for corresponding characteristics of electromagnetic wave by changes of shape and kind of metal pattern and dielectric layer have been reported until now: metamaterial using gold which shows transparent changes in THz range by changes of magnetic field^14^, metamaterial using gold which shows polarization direction changes of electromagnetic wave at 1.4 THz^15^, metamaterial using gold quantum dot which can change frequency absorbed by direction of electric field^16^, metamaterial using gold which shows improvement of resistive heat loss in solar generation^17^. Metamaterial generally has trilayer structure laminating high-conductivity patterned metal film of negative dielectric constant and non-patterned metal film, respectively, on the front side and the back side of the dielectric substrate of positive dielectric constant^18^. The metamaterial absorbers have limitations for application because of the problems that they commonly have very narrow absorption band and show dispersion phenomenon outstandingly in far-infrared domain due to metal’s own characteristics. Furthermore, there are difficulties in development of wide-band PMA which can absorb in wide frequency range, due to the narrow absorption band. To solve the problem, results of study on PMA with more than 80% of absorption in wide absorption frequency band of 6 GHz with multilayer vertical structure metal and dielectric were repeatedly formed were reported recently^19^. However, manufacturing process for multilayer vertical structure is meticulous, so there are weaknesses such as low reproducibility and high cost of manufacture. In addition, PMA of multilayer vertical structure cannot be applied to flexible material due to the thickness. To solve the problem, studies on metamaterial absorber embedded by pattern of bendy conductive high molecule^20^, metamaterial absorber controlling frequency with liquid crystal^21^ and metamaterial absorber controlling frequency with conductive grapheme layer in THz range^22^ were conducted, but there is a weakness that those metamaterials show narrow absorption band. As industrial and military demands for metamaterials are increased recently, it’s certainly inquired to develop innovative PMA manufacturing technology which satisfies both perfect absorption characteristics and transparent/bendy characteristics at the same time in wide GHz domain.

This research intends to introduce new type of water droplet-based perfect metamaterial absorber (WD-PMA) arranging water droplets on the surface of various substrates by controlling surface wettability. If examining meta-studies related to water, there were study electromagnetic wave reflection can be reduced in THz range by adding water of high extinction coefficient characteristics to metal meta-pattern^23^ and study on absorption of electromagnetic wave in GHz range by using water of dielectric characteristics as metamaterial substrate^24^. In addition, the tunable microwave photonic device was reported using by high temperature-dependent dielectric permittivity of a glass cylinder with heated water^25^. But, experimental results and theoretical analysis on development of nearly PMA which can absorb electromagnetic wave in GHz, a radar detecting domain, with meta-atoms of patterned water droplets are firstly released.

This research examined the method to control absorption frequency of electromagnetic wave in X–band (8–12 GHz) and K_U_–band (12–18 GHz) for mobile communication, satellite and radar with much practical value with patterning of simple water droplets. It also intended to confirm possibilities of active metamaterial which can change absorption band and absorption ratio by controlling height and diameter of water droplets. It also analyzed how water with low conductivity can absorb electromagnetic wave of wide band and how it can control absorption ratio theoretically. Unlike existing metamaterials based on conductive in/organic materials, metamaterial using water droplets can form and eliminate water droplets on various substrates, so it can give and eliminate easily meta-properties.

First of all, we have selected water instead of metal due to the fact that water is liquid, which means that water is in controllable shape and thickness. In addition, water has ion currents, even though low. The dielectric permittivity and the conductivity of water are set to be 78 and 1.59 S/m in CST Microwave Studio software®, respectively. However, these properties are rapidly changed in the 6–18 GHz range. Therefore, we had fitted the dielectric permittivity and the loss tangent of water using known values in Fig. 1^26^. As shown in Fig. 1, the dielectric permittivity is dramatically reduced according to frequency. In contrast, the loss tangent significantly increases. When we simulated all the absorption spectra, these values were applied to CST Microwave Studio software®. To grasp the degree of electromagnetic wave absorption by periodic existence of water droplets, the simulation was performed. The unit cell of meta-atom was set as periodic boundary conditions on the *x*–*y* plane and designed for the range of 6–18 GHz as a structure propagating electromagnetic wave in the out-of-plane direction (the *z*-axis).

The results couldn’t be embodied in metamaterial absorber using metal pattern which was previously reported and firstly showed that metamaterial absorber using water can be applied to very wide absorption band. It’s noticeable that metamaterial absorber based on metal inquires high conductivity (~10^7^ S/m) so as to develop metamaterial of excellent performance generally^18^. WD-PMA suggested by this research presented more excellent performance than existing metamaterial based on metal, even with low conductivity (less than 10 S/m) of water. To understand how water droplets of low conductivity show metamaterial effectively more deeply, we measured absorption of electromagnetic wave by height and diameter of water droplets through the experiment and compared and analyzed with simulation results. Figure 2a shows the pattern of water droplets which were periodically formed on flame retardant number 4 (FR-4: ε = 4.3 and tan δ = 0.025), the thickness of FR-4 is 2 mm. While diameter was maintained at 8 mm with the characteristics of surface wettability, we controlled 6 steps of water droplet height from 0 (status without water droplet) to 0.5, 1.0, 1.5, 2.0 and 2.5 mm. In addition, we could control 4 steps of water droplet diameter, 8, 10, 12 and 14 mm, through the hydrophobic/hydrophilic surface patterning. (refer to Section I in Supplementary : manufacturing method)

Figure 2b shows changes of electromagnetic-wave absorption spectrum from no water to high water drops. Substrate without water droplet generates weak absorption caused by dielectric loss. On the contrary, if the height is more than 0.5  mm, there is a characteristic of perfect absorber whose absorption is more than 93%. Note that perfect absorption commonly means absorption of more than 93%. It’s interesting that absorption spectrum is shifted to low frequency domain while height of water is gradually high. While height of water was heighten to be 0.5, 1.0, 1.5, 2.0 and 2.5 mm, the first frequency whose absorption is more than 93% was transferred to low frequency domain of 11.50, 10.58, 10.29, 9.62 and 7.95 GHz gradually. Especially, if thickness of water drop was 2.5 mm, more than 91% of average absorption ratio was shown in very wide range of Δ = 10 GHz. For height of 1.5 and 2 mm, two broad peaks could be observed clearly around 10 and 17 GHz, which is quite similar to the simulation in Fig. 1c. From the simulation, the resonance absorption appears at 7 peaks applying the values of CST Microwave Studio® 2011. (see Fig. S2 in Supplementary) On the other hand, two resonance peaks appears when the dielectric functions of water is considered^26^. The two wide peaks are actually composed of two or three peaks. Figure 2c presents total absorption ratio of electromagnetic wave and the first frequency generating more than 80% of absorption, which is stopband edge, by height of water droplet. Total absorption is very low as 1.56 if there is no water droplet in 8–18 GHz (Δ = 10 GHz), but is enhanced to be 7.9, 8.56, 8.62, 8.91 and 9.1 as height of water drop is increased to 0.5, 1.0, 1.5, 2.0 and 2.5 mm, respectively. Also, there is clear phenomenon of red-shift that stopband edge is transferred to low frequency domain as height of water drop is increased. Absorption of WD-PMA is generated by movement of current density in water droplets patterned periodically. As height of water is increased, the space current density can move in water droplet is increased, current path becomes long and absorption is generated in low frequency domain of long wavelength. Therefore, as height of water droplet is increased, absorption frequency is shifted to low frequency. Through the experimental results, we realized that absorption frequency can be controlled by adjusting the height of water droplet. In case of absorption ratio by height of water droplet, electromagnetic wave absorption was too small at the status height of water droplet is very thin (height 36 μm) with low electric conductivity, but it’s possible to absorb by increasing height of water droplet. Generally, if conductivity of metamaterial is low, absorption of electromagnetic wave becomes low. If examining characteristics of electromagnetic wave absorption while changing conductivity of metamaterial perfect absorber based on thin metal film from 50 to 5.8 × 10^7^ S/m, absorption ratio was clearly reduced while full width at half maximum (FWHM) became greater as the conductivity was lower. (see Fig. S3 in Supplementary) According to the results, electromagnetic-wave absorption of WD good MA is high although absorption should be low in case of low conductivity like water because movement of current density in water is increased while height of water drop is increased.

It’s necessary to examine changes of WD-PMA characteristics by not only height of water droplet but also diameter of water droplet. Figure 2d shows changes of absorption spectrum when diameter of water droplet is decreased to 14, 12, 10, and 8 mm by fixing height of water droplet as 2.0 mm (Pattern periodicity of water droplets is decreased to 20, 17, 14, and 12 mm, respectively). As the Figure shows, absorption ratio was increased and absorption range was increased while diameter of water droplet was reduced from 14 to 8 mm. Figure 2e shows correlations between total absorption quantity and stopband edge frequency in 8–18 GHz by changes of water droplet diameter. As diameter of water droplet was reduced, total absorption was increased in 8–18 GHz. Total absorption quantity by diameter of water droplet was increased to 6.55, 6.7, 8.4 and 8.91 while diameter of water was reduced to 14, 12, 10 and 8 mm, respectively. Especially, if diameter was 8 and 10 mm, average absorption was high, more than 84%, in 8–18 GHz. Stopband edge frequency was shifted to low frequency of 9.16, 8.84, 8.2 and 7.76 GHz while water diameter was reduced to 14, 12, 10 and 8 mm, respectively. In conclusion, total absorption was increased in wide band and transferred to lower-frequency absorption band while water droplet diameter was reduced and height of water droplet was increased.

Figure 3 shows experimental results for 4 substrates of different dielectric constants and mechanical characteristics, paper, polyethylene terephthalate (PET), FR-4 and glass. Figure 3b reveals experimental results for 4 substrates of different dielectric constants and mechanical characteristics, paper (ε = 2.31 and tan δ = 0.05), polyethylene terephthalate (PET : ε = 2.8 and tan δ = 0.003), FR-4 (ε = 4.3 and tan δ = 0.025), and glass (ε = 4.82 and tan δ = 0.0054) and electromagnetic wave absorption of water droplet structure formed periodically on the surface. The thickness of all dielectric substrates and the copper layer is 2 mm and 36 μm, respectively. On the surface of 4 substrates, droplets with diameter of 8 mm and height of 2 mm were formed commonly and periodically. And then, electromagnetic wave of 6–18 GHz was incident on 4 WD-PMA manufactured and absorption of electromagnetic wave was grasped with reflection wave measuring system. (refer to Section IV in Supplementary : measuring system) Substrates water droplets were formed periodically showed very high absorption of electromagnetic wave in frequency range of 6–18 GHz. It’s noticeable that perfect absorption was observed in certain frequency domain regardless of the kind of substrate.; FR-4 substrate showed perfect absorption of 93% in 8.3–12.07 GHz, PET showed in 11.23–12.36 GHz, paper showed in 9.2–16.5 GHz and glass showed in 12.05–12.65 GHz. Especially, if examining electromagnetic wave absorption in FR-4 and paper substrate, it can make perfect metamaterial absorber absorbing more than 93% of electromagnetic wave in very wide frequency range with only water droplets formed periodically.

We calculated distribution of current density, surface current, and induced magnetic field through the simulation, understood interaction between the GHz electromagnetic waves and WD-PMA and analyzed mechanism of perfect absorption (Fig. 4). Upper Figure of the center is mimetic diagram investigating electromagnetic wave vertically on meta-atom whose back side is metallic ground plate (Region C) and front side is water droplet (Region A) based on the glass substrate (Region B). Magnetic field of electromagnetic wave is paralleled by *x* axis, electric field is paralleled by *y* axis and incident electromagnetic wave is progressed in *z* direction. As the result of simulation shows, 3 main absorption peaks (peak A: 8.3 GHz, peak B: 10.3 GHz, and peak C: 12.3 GHz) revealed the gap of 2 GHz. They are fairly same as main peaks (peak A: 7.3 GHz, peak B: 9.4 GHz, and peak C: 12.3 GHz) shown by experimental results. According to the analysis on impedance matching which is well known, anti-parallel electric currents are created in two conductive layers by interaction between meta-atom and electromagnetic wave and incident electromagnetic wave is eliminated at particular frequency. In WD-PMA, anti-parallel currents were generated in two conductive layers (Region A and C) and it can be explained by impedance matching. It’s important that meta-atom of singular structure makes multiple absorption peaks unlike existing PMA in case of WD-PMA and it means current vortex is generated in water droplet, the current is polarized and incident electromagnetic wave has absorption spectrum of equal interval uniquely. Generally, ionic current of liquid in electric field induces mass transport such as water molecule and vortex is formed by boundary condition in the space locked, such as water droplet. Those characteristics can be clearly shown in results of WD-good-MA simulation (upper left and right in Fig. 4). Especially, peak A is existing as current density (J_down_) current flows downward in the center of water droplet and current density (J_up_) it flows upward. Peak C shows that number of antiparallel current modes is increased in water droplet (J_down_, J_up_, J_down_, J_up_, and J_down_). The result presents that absorption frequency is proportionally increased as the number of current modes is increased, which is explained by various resonance modes for perfect absorption^27^. In Peak B, absorption forms current vortex whose type is different from peaks A and C and more detailed analysis will be treated in Figure S5 and Video S1. On the other hand, the experimental results of peaks A and B are slightly different from the simulation ones (such as broadening and red-shift). These can be explained by the following reasons. Firstly, the dielectric properties of water are rapidly changed by the surrounding environments (temperature, etc.) in 6–18 GHz. The second reason is slight difference in the shape of water droplet between simulation and reality, since the actual shape of water droplets is affected by the gravity. Actually, deformation of water droplet induced peak shift in low frequency range more outstandingly than that through the simulation results.

There are application areas inquiring perfect absorption of electromagnetic wave for the purpose of using. Especially, military appliances, airplane, warship, tank, communication facility and central control room for military transportations and facilities shouldn’t be detected by radar of wide GHz domain. Radar generally grasps the location of material by detecting electromagnetic wave released and returned from the surroundings in GHz range. New type of perfect absorber suggested by this research is based on the concept water droplets are not grasped by radar as they perfectly absorb it in wide GHz frequency domain when arranged periodically on material surface. As simulation result of Fig. 5a shows, absorption ratio of FR-4 without water droplets was less than 20% in 6–18 GHz domain (more than 80% of incident electromagnetic wave is reflected on substrate) and it means easy detection from radar. If water cover the whole surface of material (Fig. 5b), absorption was less than 30% in 6–18 GHz domain. On the contrary, substrate that water droplets of same shape are periodically patterned (diameter, height, and period of 8, 2, and 12 mm, respectively) (Fig. 5c) exhibits two high absorption broad peaks in GHz range: 98% at 9.42 GHz and 89% at 17.3 GHz. Here, we employed the complex dielectric function of water due to the fact that there is a radical change in the permittivity of water in the GHz range^26^. To determine the polarization independence, we simulated both TE and TM polarizations. The absorption spectra of both TE and TM are coincident, since the periodic water droplets have a symmetrical arrangement. As the above-mentioned, high absorption is shown at two frequencies because electromagnetic wave incident on the water-droplet periodically-patterned substrate makes surface current of the direction opposed to ion current in water droplets and on copper surface. The induced current of anti-parallel canceled out while new induced magnetic field for the direction opposed to magnetic field of incident electromagnetic wave inside dielectric substrate was made. On the other hand, the water droplets can be influenced by the strong vibration, extreme temperature and dirty atmosphere. Water droplets are easily removed and re-set according to our manufacturing process. Therefore, if water droplets were influenced by the surrounding environments, new water was reset periodically after removing old water droplets. To overcome these problems fundamentally, in addition, the method to fill water in periodical cylindrical holes was proposed (refer to Section V in Supplementary).

It experimentally proves that wide-band good MA absorbing electromagnetic wave in 8–18 GHz can be manufactured by water droplet patterning and those reasons were analyzed by simulation. Metamaterial water droplets are formed periodically on the surface of paper, PET, FR-4 and glass, and show absorption spectrum of wide frequency domain, which cannot be seen in existing metamaterials based on metal. In addition, stopband edge could be tuned by height and diameter of water droplet. As height of water droplet was more, it was transferred to low frequency domain and as diameter of water droplet was more, it was also shifted to low frequency domain. The fact suggests possibilities of active metamaterial which can tune for cell phone communication band, satellite band or radar band by just controlling volume of water droplet in same substrate. As the result of simulation, water of very low electric conductivity makes absorption peak and broadening unlike metal pattern. The result was obtained after ion current made by electromagnetic wave in water droplet controlled the resonance frequency of induced magnetic field. In conclusion, it firstly showed possibilities of metamaterial manufacture in materials of low electric conductivity. WD-PMA introduced by this research can be applied to various substrates of flexible material and weaknesses that existing metamaterial absorber has narrow absorption band and materials of good conductivity should be used as meta-pattern can be endured, so it should be applied to various fields.

## Method Summary

### Manufacturing method of water droplet-based nearly-perfect metamaterial absorber

For patterning periodically water droplets used as metamaterial, hydrophile was given to the domain water droplets should exist on the surface of 4 dielectric substrates (paper, PET, FR-4, and glass) whose size was 200 × 300 mm and hydrophobicity was given to the domain water droplets shouldn’t exist. The details are described in Supplementary Information.

### Electromagnetic-wave absorption measurement

4 substrates (paper, PET, FR-4, and glass) water droplets with certain height and diameter were patterned periodically were installed vertically on sample holder. To prevent overlap of incident electromagnetic wave and reflected electromagnetic wave, transmitting antenna illuminating electromagnetic wave of linear polarization and receiving antenna detecting electromagnetic wave reflected from WD-good-MA specimen were installed at the position of 5^o^ based on specimen verticality, 2 m from the specimen. As transmission does not occur owing to the copper plate at the backside of substrate, *S*_*21*_, scattering parameter for electromagnetic-wave transmission, becomes zero and *S*_*11*_, scattering parameter for electromagnetic-wave reflection, can be obtained by Hewlett-Packard E8363B network analyzer related to transmitting antenna and receiving antenna.

### Electromagnetic-wave simulations

**Figure 2:**
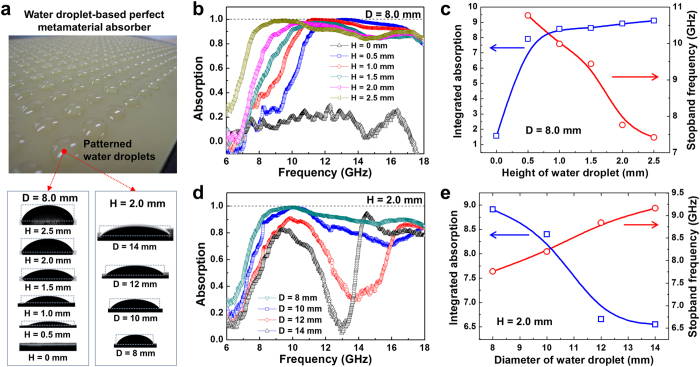
Changes of electromagnetic-wave absorption by height and diameter of water droplet. (**a**) Images of FR-4 substrate water droplet patterned periodically and contact angle images of water droplet whose height and diameter are controlled. (**b**) Changes of absorption spectrum when changing height of water droplet by 6 steps (0, 0.5, 1.0, 1.5, 2.0, and 2.5 mm) by fixing diameter of water droplet as 8.0 mm. As height of water droplet is more, absorption width of electromagnetic wave is more. (**c**) Total absorption quantity and changes of stopband edge in 8–18 GHz by changes of water droplet height. As the height of water droplet is more, total absorption quantity in 8–18 GHz (Δ = 10 GHz) is increased and stopband edge is transferred to low frequency. (**d**) Changes of absorption spectrum when diameter of water droplet is changed to 4 steps (8, 10, 12, and 14 mm) by fixing height of water droplet as 2.0 mm. As diameter of water droplet is less, absorption width of electromagnetic wave is more. (**e**) Total absorption quantity and changes of stopband edge in 8–18 GHz by changes of water droplet diameter. As diameter of water droplet is less, total absorption quantity in 8–18 GHz is increased and stopband edge is shifted to low frequency.

**Figure 3:**
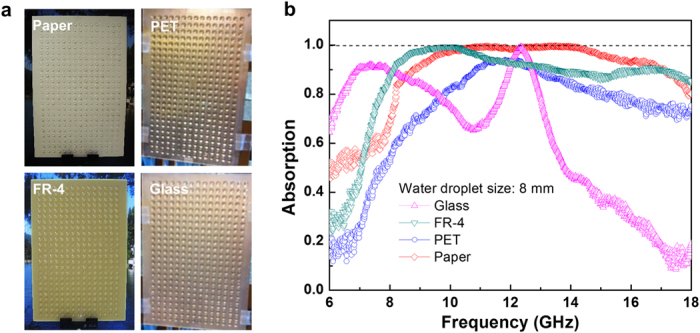
Comparison of WD-PMA characteristics by substrate. (**a**) Images that water droplets are formed periodically on the surface of substrates with 4 different dielectric constants and mechanical characteristics: paper (ε = 2.31, non-transparent flexible material), PET (ε = 2.8, transparent and flexible material), FR-4 (ε = 4.3, non-transparent rigid material), and glass (ε = 4.82, transparent rigid material). Although substrate is in vertical state, water droplets (water droplet diameter = 8 mm, height = 2 mm, pitch = 12 mm) are stably arranged without downward flow. (**b**) Electromagnetic wave absorption spectrum of manufactured 4 WD-PMA. 4 substrates with the structure of periodical water droplets show good-absorption properties in frequency range of 8–18 GHz. Absorption spectra show differences by substrate’s dielectric constants and loss tangent.

**Figure 4:**
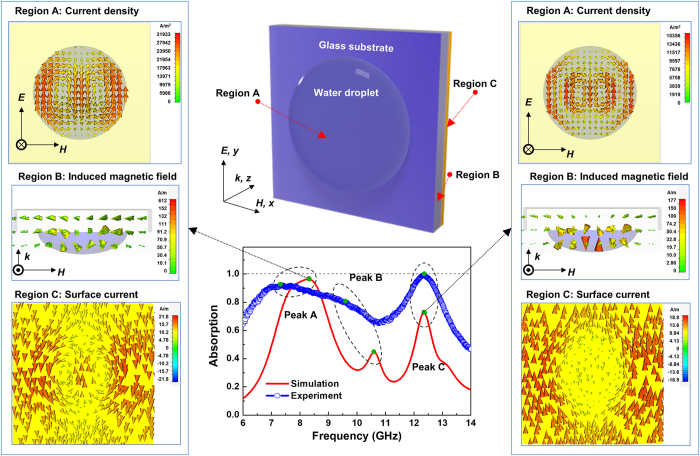
Simulation about absorption resonance phenomenon of electromagnetic wave. Upper central Figure is mimetic diagram of meta-atom organized by water droplet (Region A) of the front side and metallic ground plate (copper plate) (Region C) of the back side based on glass plate (Region B). The results of three-dimensional simulation grasping electromagnetic wave absorption root of water droplet can be obtained by taking meta-atom as unit cell and injecting electromagnetic wave of electric field (E) paralleled by *y* axis and electric field (H) paralleled by *x* axis in the direction of *k* vector paralleled by *z* axis. Central lower graph is comparison of electromagnetic wave absorption obtained by simulation and experiment. The root of two main absorption peaks, peak A (left Figure) and peak C (right Figure) can be explained by flow of current density formed in water droplet (Region A), induced magnetic field in specimen (Region B) and flow of surface current on copper plate (Region C).

**Figure 5:**
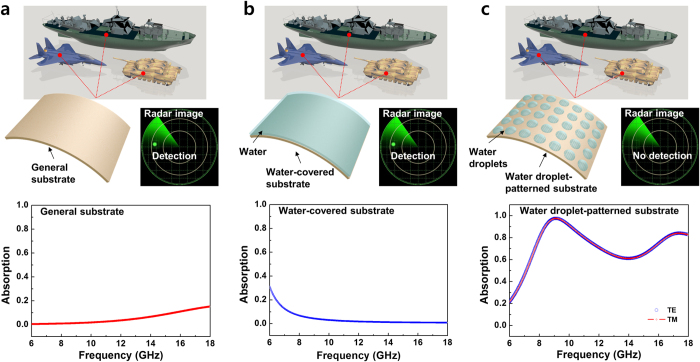
Mimetic diagram of radar detection and results of electromagnetic-wave absorption spectrum in 6–18 GHz by formation of water droplets on material surface. (**a**) Radar detection and electromagnetic wave absorption of general materials, airplane, warship, and tank. (**b**) Radar detection and electromagnetic wave absorption when water covers the whole material surface. (**c**) Radar detection and electromagnetic wave absorption when water forms certain intervals (water droplet diameter = 8 mm, height = 2 mm, pitch = 12 mm) on material surface. If water covers the whole general material and material surface, electromagnetic wave reflection is made and detected by radar. On the contrary, if water droplets patterned periodically are applied to material, perfect-absorption properties are shown in frequency range of 6–18 GHz.

The simulations in Figs 2, 3, 4, 5, Supplementary Figs S2, S5b and S5c, and Video S1 were performed using CST Microwave Studio® 2011. The simulations were performed using a frequency-domain solver.

## Additional Information

**How to cite this article**: Yoo, Y. J. *et al.* Metamaterial Absorber for Electromagnetic Waves in Periodic Water Droplets. *Sci. Rep.* **5**, 14018; doi: 10.1038/srep14018 (2015).

## Supplementary Material

Supplementary Information

Supplementary information

Supplementary information

Supplementary information

Supplementary information

## Figures and Tables

**Figure 1:**
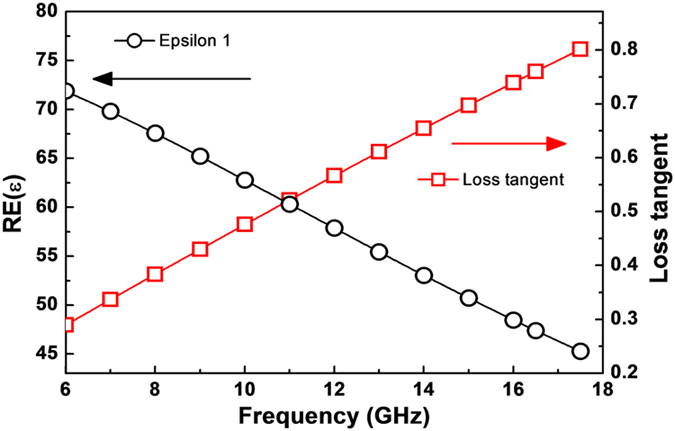
Fitted dielectric permittivity and loss tangent of water in 6–18 GHz. The dielectric permittivity and the conductivity of water are set to be 78 and 1.59 S/m in CST Microwave Studio software®, respectively. However, these values significantly differ from the real ones in the 6–18 GHz range. The dielectric permittivity is rapidly reduced by increasing frequency. On the other hand, the loss tangent significantly increases. These fitted dielectric properties were applied to CST Microwave Studio software® for all the calculations.

## References

[b1] Shelby R. A., Smith D. R. & Schultz S. Experimental verification of a negative index of refraction. Science 292, 77–79 (2001). 11292865 10.1126/science.1058847

[b2] Cheng D. *et al.* Numerical study of a dualband negative index material with polarization independence in the middle infrared regime. J. Opt. Soc. Am. B 30, 224–228 (2013).

[b3] Ebbesen T. W. *et al.* Extraordinary optical transmission through sub-wavelength hole arrays. Nature (London) 391, 667–669 (1998).

[b4] Yannopapas V., Paspalakis E. & Vitanov N. V. Electromagnetically induced transparency and slow light in an array of metallic nanoparticles. Phys. Rev. B 80, 035104 (2009).

[b5] Pendry J. B. Perfect cylindrical lenses. Opt. Express 11, 755–760 (2003). 19461787 10.1364/oe.11.000755

[b6] Pendry J. B., Schurig D. & Smith D. R. Controlling electromagnetic fields. Science 312, 1780 (2006). 16728597 10.1126/science.1125907

[b7] Schurig D. *et al.* Metamaterial electromagnetic cloak at microwave frequencies. Science 314, 977 (2006). 17053110 10.1126/science.1133628

[b8] Li L. W. *et al.* A broadband and high-gain metamaterials microstrip antenna. Appl. Phys. Lett. 96, 164101 (2010).

[b9] Liu N., Mesch M., Weiss T., Hentschel M. & Giessen H. Infrared perfect absorber and its application as plasmonic sensor. Nano Lett. 10, 2342–2348 (2010). 20560590 10.1021/nl9041033

[b10] Hao J., Zhou L. & Qiu M. Nearly total absorption of light and heat generation by plasmonic metamaterials. Phys. Rev. B 83, 165107 (2011).

[b11] Landy N. I. *et al.* Perfect metamaterial absorber. Phys. Rev. Lett. 100, 207402 (2008). 18518577 10.1103/PhysRevLett.100.207402

[b12] Liu X. *et al.* Taming the blackbody with infrared metamaterials as selective thermal emitters. Phys. Rev. Lett. 107, 045901 (2011). 21867022 10.1103/PhysRevLett.107.045901

[b13] Liu X., Starr T., Starr A. F. & Padilla W. J. Infrared spatial and frequency selective metamaterial with near unity absorbance. Phys. Rev. Lett. 104, 207403 (2010). 20867064 10.1103/PhysRevLett.104.207403

[b14] Scalari G. *et al.* Ultrastrong coupling of the cyclotron transition of a 2D electron gas to a THz metamaterial. Science 335, 1323 (2012). 22422976 10.1126/science.1216022

[b15] Grady N. K. *et al.* Terahertz metamaterials for linear polarization conversion and anomalous refraction. Science 340, 1304 (2013). 23686344 10.1126/science.1235399

[b16] Decker M. *et al.* Dual-channel spontaneous emission of quantum dots in magnetic metamaterials. Nature Comms . 4, 2949 (2013). 10.1038/ncomms3949 24335832

[b17] Vora A. *et al.* Exchanging ohmic losses in metamaterial absorbers with useful optical absorption for photovoltaics. Sci. Rep . 4, 4901 (2014). 24811322 10.1038/srep04901 PMC4014987

[b18] Pendry J. B., Holden A. J., Robbins D. J. & Stewart W. J. Magnetism from conductors and enhanced nonlinear phenomena. *IEEE Trans*. Microwave Theory Tech . 47, 2075 (1999).

[b19] Ding F. *et al.* Ultra-broadband microwave metamaterial absorber. Appl. Phys. Lett. 100, 103506 (2012).

[b20] Drexler C. *et al.* Terahertz split-ring metamaterials as transducers for chemical sensors based on conducting polymers: a feasibility study with sensing of acidic and basic gases using polyaniline chemosensitive layer. Microchim Acta 181, 1857–1862 (2014).

[b21] Shrekenhamer D., Chen W.-C. & Padilla W. J. Liquid crystal tunable metamaterial absorber. Phys. Rev. Lett. 110, 177403 (2013). 23679774 10.1103/PhysRevLett.110.177403

[b22] Andryieuski A. & Lavrinenko A. V. Graphene metamaterials based tunable terahertz absorber: effective surface conductivity approach. Opt. Express 21, 9144 (2013). 23572003 10.1364/OE.21.009144

[b23] Miyamaru F. *et al.* Reduction of effect of water absorption for metamaterial sensing applications. In: *Metamaterials’2012, The Sixth International Congress on Advanced Electromagnetic Materials in Microwaves and Optics, St. Petersburg, Russia, 345–347* (2012, 17–22 Sep), ISBN 978-952-67611-2-1.

[b24] Dong B. *et al.* An absorptive filter using microfluidic switchable metamaterials. In: *Solid-State Sensors, Actuators and Microsystems Conference, Beijing, China, 530–533 IEEE* (2011, 5–9 June), ISBN 978-1-4577-0157-3.

[b25] Rybin M. V. *et al.* Switching from visibility to invisibility via fano resonances: theory and experiment, Sci. Rep . 5, 8744 (2015). 25739324 10.1038/srep08774 PMC4350085

[b26] Meissner T. H. & Wentz F. J. The complex dielectric constant of pure and sea water from microwave satellite observations. IEEE Trans. Geos. Remote. Sens . 42, 1836–1849 (2004).

[b27] Balanis C. A. Antenna Theory: Analysis and Design , 3rd Edition. Vol. 1 Ch. 9, 510–511 and Ch. 14, 869–871 (Wiley, New Jersey, 2005).

