# Supplementary material for: Metamaterial Absorber for Electromagnetic Waves in Periodic Water Droplets
Source: Sci Rep. 2015 Sep 10;5:14018. doi: 10.1038/srep14018 (PMC4564857; doi:10.1038/srep14018)
Supplement: Supplementary Information [file srep14018-s1.pdf]

Supplementary Information for 'Metamaterial Absorber for Electromagnetic Waves in Periodic Water Droplets'

Young Joon Yoo, Sanghyun Ju, Sang Yoon Park, Young Ju Kim, Jihye Bong, Taekyung Lim, Ki Won Kim, Joo Yull Rhee, YoungPak Lee

Here, in this Supplementary Information, we present details on the manufacturing method for water droplet-based perfect metamaterial absorber, details on the electromagnetic-wave absorption measurement, simulation and experimental results for metamaterial filling water in periodical cylindrical holes, and video for current density and surface current movement.

I. Manufacturing method for water droplet-based nearly-perfect metamaterial absorber.

Figure S1 shows the mimetic diagram of WD-PMA manufacturing method and the image by substrate. For patterning water droplets used as metamaterial periodically, hydrophile was given to the domain water droplets should exist on the surface of 4 dielectric substrates (paper, PET, FR-4, and glass) whose size was 200 × 300 mm and hydrophobicity was given to the domain water droplets shouldn't exist. Figure S1a shows schematic diagram for water droplet patterning process using spray coating and O_2 plasma treatment. By adding heptadecafluorodecphosphonic acid (HDF-PA, Apollo Scientific Ltd., U. K.) to isopropyl alcohol (IPA) as the concentration of 5 mM, IPA–HDF-PA solution was made. And then, 80 g of Al_2O_3 nanoparticle (diameter of 20 nm, Sigma Aldrich, U. S. A.) was put to 100 mL of IPA–HDF-PA solution and nonication was conducted for 1 h for good self-assembly. The composed HDF-PA self-assembled Al_2O_3 solution was coated on substrate surface by the density of 0.033 g/cm^2 through the spraying method. During the spray coating, spraying power was controlled by N_2 gas quantity. Through the process, the substrate coated by HDF-PA self-assembled Al_2O_3 showed hydrophobic property (contact angle of 148.9–151.8^o). And then, O_2 plasma treatment (30 sccm, 70 W, and 30 s) was conducted by shadow mask in only domain water droplets will

be formed. After then, parts hidden by shadow mask maintain hydrophobicity and only parts treated by O_2 plasma maintain hydrophile because super-hydrophilic property is shown as carbon bonding of phosphonic acid functional group is cut by O_2 plasma[1-3]. Figure S2b shows frontal image of water droplet shape formed on paper, PET, FR-4, and glass with the thickness of 2 mm. Diameter of water droplet was controlled by the method to change the size of open area of circle shape with hydrophilic properties of shadow mask and height of water droplet was controlled by the method controlling water droplet quantity with pipet.

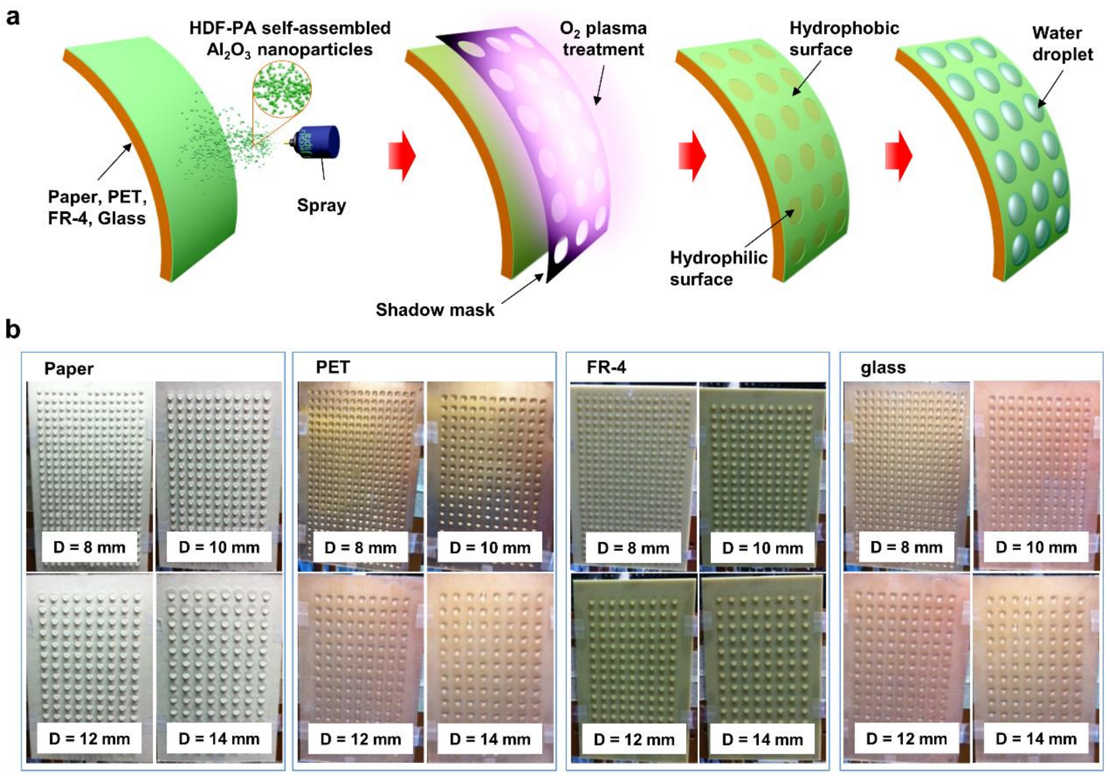

Figure S1 | Forming method of water droplet on substrate. a, Hydrophil and hydrophobic treatment related process for periodical patterning of water droplet with certain height and diameter on dielectric substrate. Through spray coating and O_2 plasma treatment of HDF-PA self-assembled Al_2O_3 nanoparticles, hydrophile was given to the domain water droplet should be remained and hydrophobicity was given to the domain water droplet should be not existing. **b,** Frontal picture of water droplet formed on the surface of 4 kinds of substrates (paper, PET, FR-4, and glass) which are vertically fixed on sample holder in anechoic chamber for measurement of electromagnetic wave

absorption. Shape of water droplet is maintained well even at the state that water droplets with 4 different diameters (D = 8, 10, 12, and 14 mm) are stood in each substrate.

II. Change of the absorption spectrum according to the dielectric constant of water.

Figure S2 shows change of the absorption spectrum according to the dielectric constant of water. When the dielectric constant ($\varepsilon = 78$) and the electric conductivity (1.6 S/m) of water were set as in CST Microwave Studio® 2011, multiple absorption peaks appear with high absorption in the 6–18 GHz frequency range. However, the dielectric constant and the dielectric loss of water is dramatically changed in this frequency range. To consider the dielectric dispersion of water in the GHz range, we set the dielectric constant and the dielectric loss of water according to Ref. 4. When the real values of water were applied to CST Microwave Studio® 2011, the absorption spectrum was broadened as in Fig. S2.

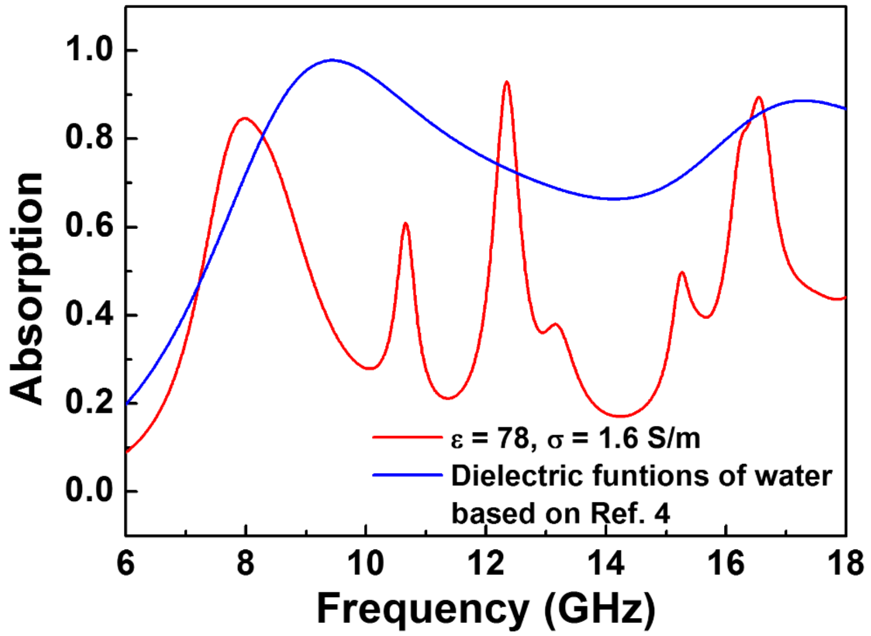

Figure S2 | Change of the absorption spectrum according to the dielectric constant of water. For the dielectric constant ($\varepsilon = 78$) and the electric conductivity (1.6 S/m) of water, several resonance peaks appear. On the contrary, when the dielectric constant and

the dielectric loss of water were set by the real values of water in the GHz range, the absorption spectrum is broadened.

III. Changes of electromagnetic-wave peak width at half height (FWHM) and absorption by metamaterial electric conductivity.

Figure S3 indicates changes of electromagnetic-wave absorption width and absorption ratio by metamaterial electric conductivity. In the Fig., as electric conductivity was reduced, absorption width of electromagnetic wave was increased and absorption ratio was reduced. When metamaterial has conductivity of general metals (~10^7 S/m), absorption is close to ~99%, but peak width at half height for absorption peak is 0.35 GHz and absorption band is very narrow. On the contrary, in case of metamaterial with very low electric conductivity (~50 S/m), absorption ratio was low as about 33%, but peak width at half height for absorption peak was 2.7 GHz greater than metal metamaterial whose electric conductivity is ~10^7 S/m by more than 7 times. It shows that absorption width of electromagnetic wave can be maximized if using metamaterial with low electric conductivity like water. The absorption ratio can be increased by increasing height of water droplets. The result shows that existing limitations for using, the electric conductivity of meta-pattern should be high, can be endured.

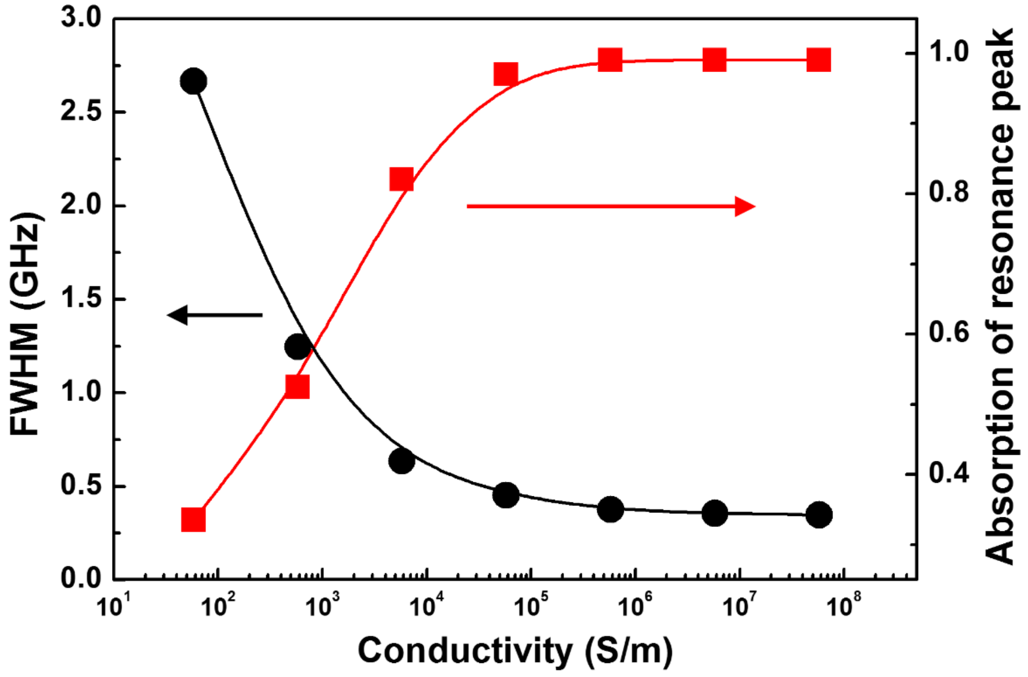

Figure S3 | Changes of electromagnetic-wave peak width at half height (FWHM) and absorption by metamaterial electric conductivity. In case of metamaterial with high electric conductivity (~10^7 S/m), peak width at half height was 0.35 GHz and absorption was ~99%. On the contrary, metamaterial of low electric conductivity (~50 S/m), FWHM was 2.7 GHz and absorption was ~33%.

IV. Electromagnetic-wave absorption measuring system.

4 substrates (paper, PET, FR-4, and glass) water droplets with certain height and diameter were patterned periodically were installed vertically on sample holder. To prevent overlap of incident electromagnetic wave and reflected electromagnetic wave, transmitting antenna illuminating electromagnetic wave of linear polarization and receiving antenna detecting electromagnetic wave reflected from WD-good-MA specimen were installed at the position of 5° based on specimen verticality, 2 m from the specimen. Electromagnetic wave absorption was found by $A(\omega) = 1 - T(\omega) - R(\omega) = 1 - (S_{21})^2 - (S_{11})^2$. In here, *A* is absorption, *T* is transmission and *R* is reflection. As transmission is not occurred owing to the copper plate at the backside of substrate, S_{21}, scattering parameter for electromagnetic wave transmission, becomes zero and S_{11}, scattering parameter for electromagnetic wave reflection, can be obtained by Hewlett-Packard

E8363B network analyzer related to transmitting antenna and receiving antenna. At that time, electromagnetic wave of linear polarization was normalized by reflected detection on conductive copper-plate sample whose size was same as the specimen.

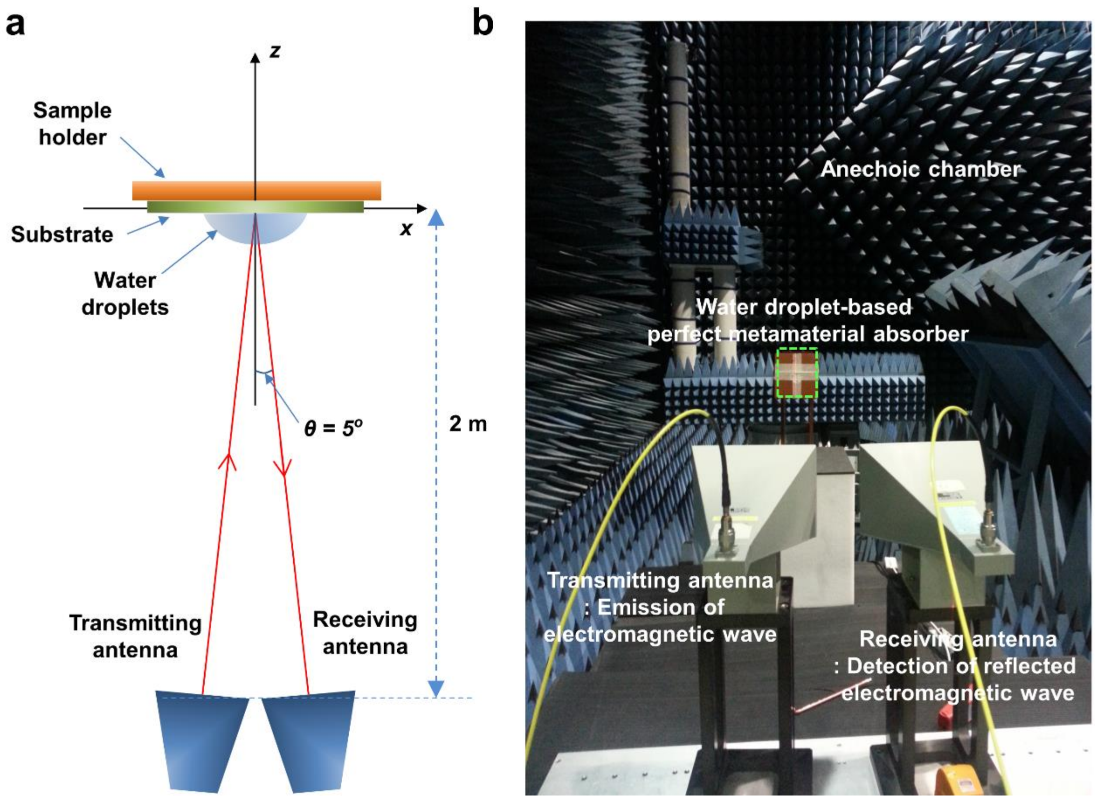

Figure S4 | Mimetic diagram and picture for electromagnetic-wave absorption measuring system. a, Mimetic diagram measuring electromagnetic-wave absorption at the status WD good MA is loaded. Electromagnetic wave absorption was measured while one of two horn antennas radiated electromagnetic wave and the other one got reflected electromagnetic wave. By attaching copper plate on back side of WD good MA, penetration was zero and incidence angle (θ) was set by 5° so as to prevent overlap of incidence wave and reflection wave. **b,** Actual photo measuring electromagnetic-wave absorption of WD good MA in anechoic chamber.

V. Simulation and experimental results for metamaterial filling water in periodical cylindrical holes.

Figure S5a is nearly-perfect MA formed by cylindrical shape after making holes in dielectric substrate and meta-atom is organized by dielectric substrate filling water, water of cylindrical shape and backside metal plate. Dielectric substrate used FR-4 of 4 mm thickness, cylindrical water's cycle was 20 mm and water's diameter was 14 mm and water's height was 2 mm. As Figure S5b shows, experimental results and simulation results are same each other in 3.5–12.4 GHz range. Particularly, absorption of 99.4% at 5.0 GHz, 99.5% at 8.33 GHz, 93.8% at 9.73 GHz, 85% at 10.74 GHz, and 72.9% at 11.9 GHz peaks in experimental results were matched with simulation results well. To examine the root of electromagnetic wave absorption in metamaterial absorber using cylindrical water, we analyzed current density in water, surface current on copper plate and induced magnetic field in specimen (Fig. S5c). Movement of water current density at main frequencies, showing electromagnetic wave absorption, was examined. As the result, absorption of 5.09 GHz showed the first eddy phenomenon in water, 7.28 GHz showed the second eddy phenomenon, 9.54 GHz showed the third eddy phenomenon and 11.65 GHz showed the fourth eddy phenomenon. On the contrary, at 8.33 and 10.62 GHz which are medium frequency domain, current density was rotated upward and downward because antiparallel surface current flows on copper plate in comparison with movement of current density in water. Therefore, induced magnetic field in the direction opposed to electromagnetic wave incident in specimen is formed and absorption is made. For improvement of understanding, video of current density and surface current at two frequencies is also prepared in Video S1.

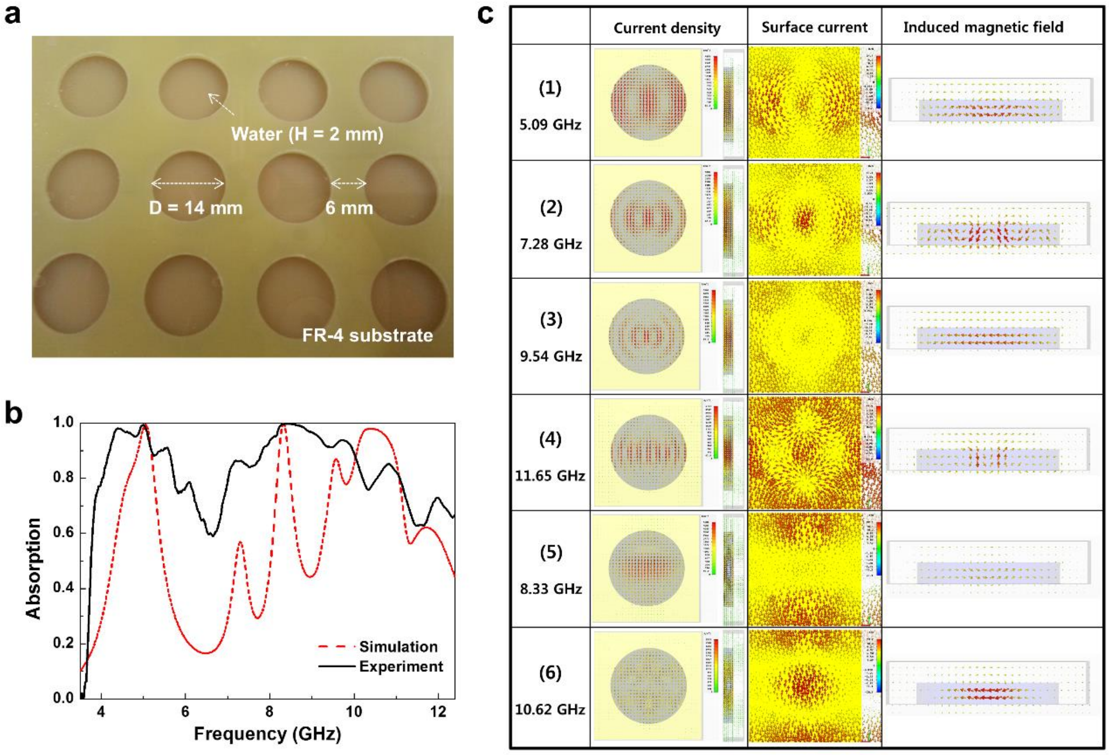

Figure S5 | Metamaterial filling periodical cylindrical holes. a, Actual appearance of specimen filling water cylindrically in FR-4 substrate. (cylindrical water's cycle = 20 mm, diameter = 14 mm, height = 2 mm) **b,** Results of comparison between simulation for absorption by frequency and experimental value. **c,** Three-dimensional image for current density of cylindrical water at main frequencies making absorption, surface current on copper plate and movement of induced magnetic field in specimen.

VI. Video for current density and surface current movement.

Video S1a and S1c show movement of current density in water droplet during the incidence of 8.3 GHz and 12.3 GHz electromagnetic wave. According to the incident electromagnetic wave, current density in water is arranged and transferred and eddy phenomenon is generated. As video shows and main body mentioned, the first eddy phenomenon and the second eddy phenomenon appear, respectively, at 8.3 and 12.3 GHz. Video shown in S1b and S1d shows movement of surface current in copper plate during the incidence of 8.3 GHz and 12.3 GHz electromagnetic wave. If watching video, movement of current density in water droplet and antiparallel surface current are formed. In conclusion, current density and surface current in

WD-PMA form induced magnetic field and induce absorption of electromagnetic wave incident on WD-PMA.

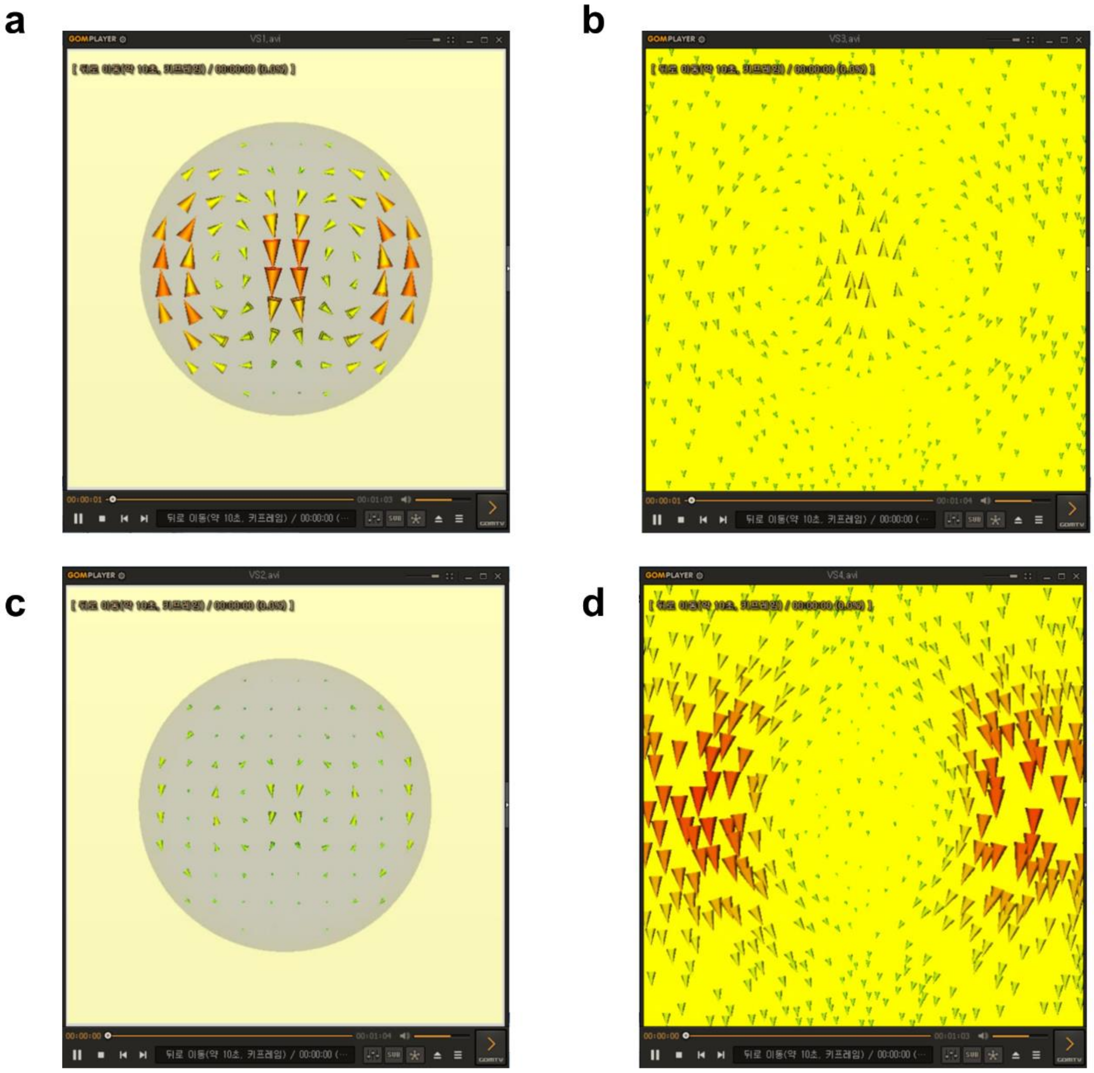

Video S1 | Video for current density and surface current movement. a, During the incidence of 8.3 GHz electromagnetic wave, movement of current density in water droplet. **b,** During the incidence of 8.3 GHz electromagnetic wave, movement of surface current in cooper plate. **c,** During the incidence of 12.3 GHz electromagnetic wave, movement of current density in water droplet. **d,** During the incidence of 12.3 GHz electromagnetic wave, movement of surface current in copper plate.

References

1. Tsougeni, K., Vourdas, N., Tserepi, A., Gogolides, E., Cardinaud, C. Mechanisms of oxygen plasma nanotexturing of organic polymer surfaces: from stable super hydrophilic to super hydrophobic surfaces. *Langmuir* **25**, 11748 (2009).

2. Kuang, P., Lee, J.-H., Kim, C.-H., Ho, K.-M., Constant, K. Improved surface wettability of polyurethane films by ultraviolet ozone treatment. *J. Appl. Polymer Sci.* **118**, 3024 (2010).

3. Sanchis, M. R., Calvo, O., Fenollar, O., Garcia, D., Balart, R. Surface modification of a polyurethane film by low pressure glow discharge oxygen plasma treatment. *J. Appl. Polymer Sci.* **105**, 1077 (2007).

4. Meissner, Th., Wentz, F. J. The complex dielectric constant of pure and sea water from microwave satellite observations. *IEEE Trans. Geos. Remote. Sens.* **42**, 1836–1849 (2004).
